# *Orthoflavivirus nilense* surveillance in the State of Piauí, northeastern Brazil

**DOI:** 10.1590/0074-027602402180

**Published:** 2025-07-07

**Authors:** Osmaikon Lisboa Lobato, Tayná da Silva Nogueira, Tobias Emílio Tavares Lima, Felipe José da Costa Andrade, Marília Gabryelle Guimarães de Macedo, Rayane de Souza Pereira, Joilson Xavier, Mariene Ribeiro Amorim, Priscilla Paschoal Barbosa, Alex Sobrinho da Rocha, Silvokleio da Costa Silva, Luiz Carlos Junior Alcantara, William M de Souza, José Luiz Proenca-Modena, Érica Azevedo Costa, Adelino Soares Lima, Lauro César Soares Feitosa, Maria do Socorro Pires e Cruz, Silvana Maria Medeiros de Sousa Silva, Silvia de Araújo França Baêta, Marcelo Adriano da Cunha e Silva Vieira, Sharon L Deem, Lilian Silva Catenacci

**Affiliations:** 1 Universidade Federal do Piauí, Centro de Ciências Agrárias, Teresina, PI, Brasil; 2 Universidade Federal do Piauí, Programa de Pós-Graduação em Tecnologias Aplicadas a Animais de Interesse Regional, Teresina, PI, Brasil; 3 Universidade Federal do Piauí, Bom Jesus, PI, Brasil; 4 Universidade Federal do Oeste da Bahia, Programa de Pós-Graduação em Ciências Ambientais, Barreiras, BA, Brasil; 5 Universidade Federal de Minas Gerais, Belo Horizonte, MG, Brasil; 6 Universidade Estadual de Campinas, Instituto de Biologia, Departamento de Genética, Evolução, Microbiologia e Imunologia, Campinas, SP, Brasil; 7 Fundação Oswaldo Cruz-Fiocruz, Instituto René Rachou, Belo Horizonte, MG, Brasil; 8 University of Texas Medical Branch, Department of Microbiology and Immunology, Galveston, TX, USA; 9 University of Texas Medical Branch, World Reference Center for Emerging Viruses and Arboviruses, Galveston, TX, USA; 10 Laboratório Central de Saúde Pública do Piauí, Teresina, PI, Brasil; 11 Instituto de Doenças Tropicais Natan Portella, Teresina, PI, Brasil; 12 Institute for Conservation Medicine, Saint Louis Zoo, St Louis, Missouri, USA; 13 Centro de Inteligência em Agravos Tropicais Emergentes e Negligenciados, Teresina, PI, Brasil; 14 Universidade Federal do Pará, Programa de Pós-Graduação em Saúde Animal na Amazônia, Castanhal, PA, Brasil

**Keywords:** flavivirus, epidemiological surveillance, zoonoses, West Nile virus

## Abstract

**BACKGROUND:**

The cycle of the *Orthoflavivirus nilense* (West Nile virus - WNV) involves birds and mosquitoes, while humans and equids serve as terminal hosts. In 2014, the first human case in Brazil was confirmed in Piauí State.

**OBJECTIVES:**

To investigate the presence of WNV in birds, mosquitoes, and equids in municipalities of Piauí.

**METHODS:**

Collections were carried out following recommendations from the Ministry of Health of Brazil, in 11 municipalities (all with human cases or bird mortality), where biological samples were collected from birds, mosquitoes, and equids. The Viral RNA extraction was performed using a commercial kit, following the manufacturers’ recommendations; samples were subjected to reverse transcription and polymerase chain reaction, with specific primers for WNV.

**FINDINGS:**

2,706 samples were collected (636 birds, belonging to 99 species; 420 equids, and 1,650 mosquitoes, grouped into 346 pools, totaling 18 species. No collected sample yielded a positive result, corroborating with other studies showing the difficulty of molecular detection of WNV in healthy animals, which may explain the non-detection, in addition to the delayed diagnosis in humans.

**MAIN CONCLUSIONS:**

A local investigation involving suspected cases is still recommended in animals; however, in locations with late diagnosis in humans we suggest a serological survey of asymptomatic birds and equids.

The *Orthoflavivirus nilense* (West Nile virus - WNV), a member of the *Flaviviridae* family, *Orthoflavivirus* genus, primarily infects mammals and birds. Humans and equines may serve as dead end hosts. The virus was detected in the Americas at the end of the 20th century, and was first discovered in Brazil in 2005.^1^ ^,^ ^2^ ^,^ ^3^ ^,^ ^4^ ^,^ ^5^ The first human case in Brazil was reported in 2014 in Piaui State, northeast Brazil^4^ and up until 2022 all WNV human cases in Brazil were confirmed in this state. Molecular detection has previously been performed in equines,^5^ ^,^ ^6^ ^,^ ^7^ ^,^ ^8^ ^,^ ^9^ but the vectors and birds involved in the epidemiological cycle in Brazil remain largely unknown. Therefore, the aim of this study was to investigate the presence of the WNV in birds, equids, and mosquito vectors in municipalities where human cases and/or unexplained mortality of free-living birds have been reported in Piauí State.

## SUBJECTS AND METHODS

*Study area and characterization of Orthoflavivirus nilense surveillance in Piauí* - A total of 11 municipalities were sampled between the years 2019 and 2022, from north to the south Piauí State, Brazil: Água Branca, Amarante, Barro Duro, Bom Jesus, Buriti dos Montes, Juazeiro do Piauí, Lagoa Alegre, Parnaíba, Piripiri, Teresina, and Valença do Piauí. These municipalities were selected based on the confirmation of human cases for WNV or high mortality of free-living birds (Figure).

**Figure:**
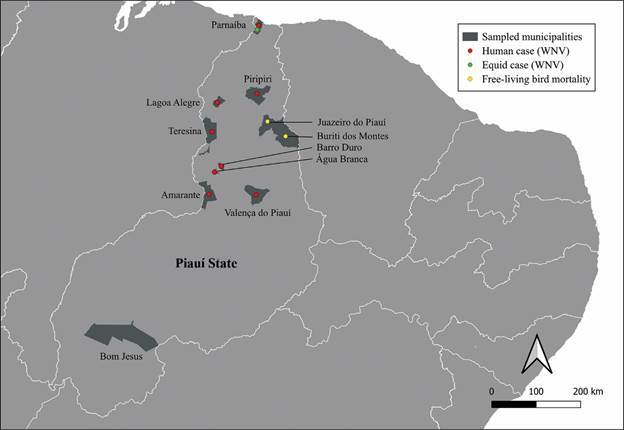
Municipalities sampled in the State of Piauí, between October 2019 and August 2022. Bom Jesus was the only state without confirmed or suspected human cases of West Nile virus (WNV) and without reports of free-living bird mortalities.

*Capture and collection of biological material* - The collection of bird, equid and mosquito samples followed the Epizootic Surveillance Guide of the Brazilian Ministry of Health,^10^ and recommendations from Tolsá et al.^11^ For bird collection, ornithological capture nets (mist nets) were employed,^12^ and birds were identified according to published guides;^13^ ^,^ ^14^ ^,^ ^15^ up to 500 µL of blood was collected from the brachial vein using microhematocrit tubes.^2^ ^,^ ^11^ In equids, we collected up to 20 mL of blood from the jugular vein of each animal. The animals were identified by species, sex, and age, and an informed consent form was provided to the owner, granting permission for the collection. Mosquitoes were collected in all municipalities, except in Bom Jesus, Buriti dos Montes, and Juazeiro do Piauí. CDC light traps were used for collection,^16^ identification was conducted following Consoli et al. and Forattini,^17^ ^,^ ^18^ and pools were formed with up to 15 mosquitoes of the same species per collection point. All collected samples were refrigerated in liquid nitrogen until laboratory processing.

Molecular biology tests for viral detection

*Bird and mosquito samples* - Genetic material extraction was carried out using silica column kits (Bioclin^®^) and magnetic beads (Zymo^®^), following the manufacturer’s recommendations. The samples subjected to a reverse transcription reaction following Kuno and Catenacci et al.,^19^ ^,^ ^20^ and the polymerase chain reaction (PCR) following Lanciotti et al.^21^ The reaction products were observed following electrophoresis on a 1% agarose gel stained with ethidium bromide.

*Equid samples* - Extraction of genetic material was performed using magnetic bead kits (Zymo^®^). For real-time PCR (RT-PCR), the samples were grouped into pools, each containing 10 distinct samples from the same location. A set of specific primers was used to amplify the complete viral genome of the WNV.^5^ The PCR result was observed using an E-Gel (Thermo Fisher^®^).

*Ethical considerations* - The research complied with the relevant laws governing research conducted in Brazil. Approval for the study was obtained from the Ethics Committee for the Use of Animals in Research of the Federal University of Piauí (CEUA/UFPI, Approval No. 605/19). Additionally, authorization was granted by the Institute Chico Mendes de Conservação da Biodiversidade (ICMBio), a division of the Brazilian Environmental Agency (ICMBio-SISBIO, Permit No. 75734-1), and the National System for the Management of Genetic Heritage and Associated Traditional Knowledge (SISGEN, Registration No. AF40BCA).

## RESULTS AND DISCUSSION

Between October 2019 and August 2022, we collected a total of 2,706 samples, including 636 birds belonging to 99 species, 420 equids, including donkeys, mules, and horses, and 1,650 mosquitoes grouped into 346 pools, totaling 18 species (Table I). All 2,706 samples underwent reverse transcription followed by WNV RT-PCR testing, but none yielded positive results. All the procedures followed the recommendations of the Ministry of Health of Brazil, both for the diagnosis and for the epidemiological surveillance of birds, equids, and vectors.^22^

**TABLE I:** Number of animals collected in each municipality

Municipalities	AB	AM	BD	BJ	BM	JP	LA	PA	PI	TE	VA	Total
Birds	62	56	47	26	35	30	63	76	64	126	51	636
Equids	48	48	54	-	-	-	45	65	60	57	43	420
Mosquitoes^*^	31	7	1	-	-	-	28	12	53	214	-	346
Total	141	111	102	26	35	30	136	153	177	397	94	1,402

TE: Teresina; PI: Piripiri; LA: Lagoa Alegre; AB: Água Branca; AM: Amarante; PA: Parnaíba; VA: Valença; BD: Barro Duro; BJ: Bom Jesus; JP: Juazeiro do Piauí; BM: Buriti dos Montes. ^*^Value refers to the mosquito pool.

The most well-represented bird families were Thraupidae (24.69%; n = 157), Phasianidae (11.79%; n = 75), and Columbidae (11.79%; n = 75) (Table II). Despite the sampling effort and the diversity of birds collected, only 3.3% considered potential sentinels for WNV based on previous studies with confirmed detection:^23^ ^,^ ^24^ *Passer domesticus* (Passeridae), with 13 specimens sampled (Água Branca n = 6, Teresina n = 6, and Amarante n = 1); *Cyanocorax cyanopogon* (Corvidae), with four specimens sampled (Valença n = 2, Bom Jesus n = 1, and Piripiri n = 1); *Glaucidian brasilianum* (Strigidae), with two specimens sampled (Piripiri n = 1, Valença n = 1); *Megacops choliba* (Strigidae), with one (1) specimen collected (Barro Duro); and *Rupornis magnirostris* (Accipitridae), with one (1) specimen collected (Piripiri). Based on the lists of dead birds detected with WNV in the United States,^25^ we can also add the Anatidae family, the most abundant among the 332 species reported; only one representative of the family was collected in Piauí: *Anas platyrhynchos domesticus*, with five specimens, all collected in Barro Duro, which does not significantly alter the percentage of potential sentinels collected in the state, which would remain below 5%.

**TABLE II:** Bird species captured during investigation of the *Orthoflavivirus nilense* circulation in the State of Piauí, Brazil, during the period from October 2019 to August 2022

Municipalities	AB	AM	BD	BJ	BM	JP	LA	PA	PI	TE	VA	Total
Thraupidae	2	14	3	2	23	23	8	30	13	23	16	157
Phasianidae	8	9	9	-	-	-	9	20	-	12	8	75
Columbidae	18	0	7	2	7	5	5	5	4	19	3	75
Tyrannidae	15	8	8	1	3	0	7	1	9	7	3	62
Furnariidae	6	0	3	1	0	1	2	0	9	25	4	51
Icteridae	0	7	0	0	0	0	20	0	1	0	0	28
Turdidae	0	1	4	12	0	0	0	0	3	1	1	22
Troglodytidae	4	1	1	1	0	0	6	0	0	4	3	20
Scolopacidae	0	0	0	0	0	0	0	17	0	0	0	17
Rhynchocyclidae	0	4	0	0	0	0	1	0	3	2	3	13
Passeridae	6	1	0	0	0	0	0	0	0	6	0	13
Thamnophilidae	0	3	0	0	0	0	0	0	0	8	1	12
Cuculidae	0	2	0	0	0	0	0	0	5	2	1	10
Dendrocolaptidae	2	0	0	1	0	0	0	0	2	2	0	7
Galbulidae	0	1	2	0	0	1	0	0	2	1	0	7
Parulidae	0	2	1	1	0	0	2	0	0	2	0	8
Passerellidae	0	2	1	0	0	0	0	0	3	0	0	6
Alcedinidae	0	0	0	0	0	0	0	2	2	1	0	5
Anatidae	0	0	5	0	0	0	0	0	0	0	0	5
Polioptilidae	0	0	0	0	0	0	0	0	1	4	0	5
Ardeidae	0	0	1	0	0	0	0	0	2	1	0	4
Corvidae	0	0	0	1	0	0	0	0	1	0	2	4
Bucconidae	0	1	0	0	0	0	0	0	0	0	2	3
Caprimulgidae	1	0	0	0	0	0	0	0	0	2	0	3
Strigidae	0	0	1	0	0	0	0	0	1	0	1	3
Estrildidae	0	0	0	0	0	0	0	0	0	2	0	2
Mimidae	0	0	0	1	0	0	0	0	0	0	1	2
Picidae	0	1	0	0	0	0	0	0	0	0	0	1
Vireonidae	0	0	0	0	0	0	2	0	0	0	0	2
Accipitridae	0	0	0	0	0	0	0	0	1	0	0	1
Charadriidae	0	0	0	0	0	0	0	1	0	0	0	1
Hirundinidae	0	1	0	0	0	0	0	0	0	0	0	1
Jacanidae	0	0	0	0	0	0	0	0	1	0	0	1
Pipridae	0	0	0	0	0	0	0	0	1	0	0	1
Rallidae	0	0	0	1	0	0	0	0	0	0	0	1
Not identified	0	0	1	2	2	0	0	0	0	1	2	8
Total	62	58	47	26	35	30	62	76	64	125	51	636

TE: Teresina; PI: Piripiri; LA: Lagoa Alegre; AB: Água Branca; AM: Amarante; PA: Parnaíba; VA: Valença; BD: Barro Duro; BJ: Bom Jesus; JP: Juazeiro do Piauí; BM: Buriti dos Montes.

However, 6.1% (n = 6) of bird species collected in this study were previously reported with antibodies to WNV in Brazil:^26^ ^,^ ^27^ ^,^ ^28^ ^,^ ^29^ 76 specimens of *Gallus gallus domesticus* (Água Branca n = 8, Amarante n = 9, Barro Duro n = 9, Lagoa Alegre n = 9, Parnaíba n = 20, Teresina n = 12, and Valença n = 8), 38 specimens of *Columbina talpacoti* (Água Branca n = 16, Barro Duro n = 7, Lagoa Alegre n = 2, Piripiri n = 4, Teresina n = 7, and Valença n = 2), 15 specimens of *Furnarius figulos* (Água Branca n = 5, Barro Duro n = 1, Bom Jesus n = 1, Lagoa Alegre n = 1, Piripiri n = 3, Teresina n = 2, and Valença n = 2), 13 specimens of *Passer domesticus* (Água Branca n = 6, Teresina n = 6, and Amarante n = 1), five specimens of *Coryphospingus pileatus* (Lagoa Alegre), and one (1) specimen of *Arenaria interpres* (Parnaíba).

Among the potential wild bird hosts for WNV collected, all have a wide distribution across Brazil. *Passer domesticus*, for example, is an exotic bird present throughout the Americas, with records of occurrence in all Brazilian states, generally associated with anthropogenic environments and highly adaptable.^30^ ^,^ ^31^ The species *C. cyanopogon*, on the other hand, is one of the eight species of corvids that occur in Brazil, mainly related to the Northeast region but also present in the North and Central-West regions.^32^ ^,^ ^33^ The representatives of the Strigidae family collected have different characteristics. *Glaucidian brasilianum* has a diurnal habit and is the largest representative of its genus in Brazil; its distribution, according to occurrence records, is more associated with the Northeast region, while *M. chiliba* has a nocturnal habit.^34^ ^,^ ^35^ Its distribution is similar to that of *P. domesticus*, but it prefers areas with more vegetation cover and is a species difficult to spot.^36^ ^,^ ^37^ Meanwhile, *Rupornis magnirostris*, the only species of the Accipitridae family, is considered the largest hawk in Brazil, with a wide distribution across the Americas, mainly in the South; it is present throughout the country, inhabiting open areas (with sufficient tree cover), forest edges, and urbanized environments.^38^ ^,^ ^39^

Regarding the characteristics and distribution of the birds collected that have already been reported with WNV exposure,^26^ ^,^ ^27^ ^,^ ^28^ ^,^ ^29^ we have *Columbina talpacoti*, distributed throughout the Cerrado, being a typical species of the Cerrado and could be considered a bioindicator of environmental quality due to its abundance in areas with little anthropogenic alteration;^40^ ^,^ ^41^ *F. figulos*, an endemic species of Brazil;^42^ *Coryphospingus pileatus*, found in South America, in countries like Venezuela, Colombia, and French Guiana, in addition to Brazil, in the Northeast, Central-West, and Southeast regions;^43^ and *Arenaria interpres* is one of the few migratory species collected, nesting in the far north of North America, Europe, and Asia, migrating to the southern Hemisphere and almost all of the Southern Hemisphere, with reports of occurrence in all regions of Brazil.^44^ The migration of birds in Brazil, coming from the Northern Hemisphere, is one of the most accepted hypotheses for the introduction of WNV in the country, since the WNV epidemiological cycle has not yet been elucidated in the country. Thus, it is still unknown which vectors and hosts exist for the virus in Brazilian territories.^45^ ^,^ ^46^ Therefore, there is great importance and necessity for investigating bird populations, vectors, and equids, whether through direct or indirect diagnostic techniques, in order to understand the transmission dynamics and the main agents involved.

Among the sampled equids, 76.67% (n = 322) were horses, followed by 9.76% donkeys (n = 41), and 5.71% mules (n = 24) (Table III). These species are considered excellent sentinels for WNV, since they often precede human cases.^6^ ^,^ ^47^ ^,^ ^48^ Unlike birds, there are reports of WNV circulation in equids, both by serological detection^26^ ^,^ ^27^ ^,^ ^49^ ^-^ ^52^ and molecular methods^6^ ^,^ ^7^ ^,^ ^8^ ^,^ ^9^ ^,^ ^29^ in the State of Piauí.^5^

**TABLE III:** Equid species physically contained during investigation of the circulation of the *Orthoflavivirus nilense* in the State of Piauí, Brazil, during the period from October 2019 to August 2022

Municipalities	AB	AM	BD	LA	PA	PI	TE	VA	Total
*Equus caballus* (Horse)	46	28	29	37	65	56	26	35	322
*Equus asinus* (Donkey)	-	3	19	5	-	-	13	1	41
Mule	-	6	3	2	-	4	8	1	24
Not identified	2	11	3	1	-	-	10	6	33
Total	48	48	54	45	65	60	57	43	420

TE: Teresina; PI: Piripiri; LA: Lagoa Alegre; AB: Água Branca; AM: Amarante; PA: Parnaíba; VA: Valença; BD: Barro Duro.

Regarding vectors, all mosquito pools belonged to the potential vector of WNV: mosquitoes in the Culicidae family. The genera *Culex* spp. (47.40%, n = 164), *Mansonia* spp. (20.81%; n = 72), *Coquillettidia* spp. (9.25%; n = 32), *Aedes* spp. (8.67%; n = 30), and *Anopheles* spp. (7.23%; 25) were the most sampled (Table IV). There is still much to explore regarding potential WNV vectors in Brazil. There remains just one confirmed detection of WNV in vectors in Brazil from the Amazon region, and identified as a mosquito in the *Culex* genus,^53^ which was the most well-represented in the present study.

**TABLE IV:** Mosquito pools sampled during investigation of the *Orthoflavivirus nilense* circulation in the State of Piauí, Brazil, during the period from October 2019 to August 2022

Municipalities	AB	AM	BD	LA	PA	PI	TE	Total
*Culex (Culex)* sp.	6	1	-	15	6	13	123	164
*Mansonia* sp.	13	3	1	-	4	12	39	72
*Anopheles* sp.	3	2	-	1	-	9	10	25
*Coquillettidia (Rhynchotaenia)* sp.	6	-	-	1	-	4	7	18
*Aedes scapularis*	1	1	-	4	-	7	4	17
*Coquillettidia (Rhynchotaenia) venezuelensis*	-	-	-	-	-	2	9	11
*Uranotaenia* sp.	1	-	-	-	-	-	7	8
*Culex (Melanoconion)* sp.	-	-	-	3	1	3	-	7
*Aedes (Stegomyia) aegypti*	-	-	-	-	-	-	5	5
*Uranotaenia hystera*	-	-	-	3	-	1	-	4
*Aedes albopictus*	-	-	-	-	-	1	2	3
*Aedes* sp.	-	-	-	-	-	-	3	3
*Coquillettidia (Rhynchotaenia) nigricans*	-	-	-	-	-	-	3	3
*Aedes serratus*	-	-	-	-	-	1	1	2
*Aedeomyia* sp.	-	-	-	-	1	-	-	1
*Haemagogus (Haemagogus)* sp.	-	-	-	1	-	-	-	1
*Limatus* sp.	-	-	-	-	-	-	1	1
*Wyeomya* sp.	1	-	-	-	-	-	-	1
Total	31	7	1	28	12	53	214	346

TE: Teresina; PI: Piripiri; LA: Lagoa Alegre; AB: Água Branca; AM: Amarante; PA: Parnaíba; BD: Barro Duro.

Despite the extensive effort in sample collection, the non-detection of WNV in this study may be related to delayed diagnosis between human infections in the State of Piauí and the study (up to four years).

*In conclusion* - We strongly recommend carrying out local surveys in animals and vectors at the time of any suspected WNV infections in humans. Additionally, in case of a delay between a WNV diagnosis in humans, serological tests should be performed in animals instead of molecular tests, as antibodies will better inform of viral circulation as a monitoring tool. We also suggest that free-living birds with neurological symptoms rescued from wildlife rehabilitation centers be tested as part of any survey, as this will increase the sample size of potential natural hosts of WNV in Brazil. In addition to understanding the importance of using official notification systems, which can lead to early detection of outbreaks, we recommend the development of mitigation strategies, both of which will be strengthened through the collaboration between different government and health sectors in the prevention of zoonoses. Lastly, we recommend testing of any neurological equids that were negative on rabies testing since WNV should be a differential diagnosis.^54^ ^,^ ^55^ ^,^ ^56^

## References

[1] de Castro-Jorge LA, Siconelli MJL, Ribeiro BDS, de Moraes FM, de Moraes JB, Agostinho MR (2019). West Nile virus infections are here Are we prepared to face another flavivirus epidemic?. Rev Soc Bras Med Trop.

[2] Figueiredo LTM (2019). West Nile virus infection in Brazil. Rev Soc Bras Med Trop.

[3] Lorenz C, de Azevedo TS, Chiaravalloti-Neto F (2022). Impact of climate change on West Nile virus distribution in South America. Trans R Soc Trop Med Hyg.

[4] Vieira MACS, Romano APM, Borba AS, Silva EVP, Chiang JO, Eulálio KD (2015). Case report West Nile virus encephalitis: the first human case recorded in Brazil. Am J Trop Med Hyg.

[5] Costa ÉA, Giovanetti M, Catenacci LS, Fonseca V, Aburjaile FF, Chalhoub FLL (2021). West Nile virus in Brazil. Pathogens.

[6] De Siqueira RF, Hansen VS, Martins MFM, Leal MLR, Bondan EF (2022). Infecção pelo vírus da febre do Nilo Ocidental em equinos no Estado de São Paulo. Acta Sci Vet.

[7] Martins LC, da Silva EVP, Casseb LMN, da Silva SP, Cruz ACR, Pantoja JAS (2019). First isolation of West Nile virus in Brazil. Mem Inst Oswaldo Cruz.

[8] Siconelli MJL, Jorge DMM, de Castro-Jorge LA, Fonseca-Júnior AA, Nascimento ML, Floriano VG (2021). Evidence for current circulation of an ancient West Nile virus strain (NY99) in Brazil. Rev Soc Bras Med Trop.

[9] Silva ASG, Matos ACD, da Cunha MACR, Rehfeld IS, Galinari GCF, Marcelino SAC (2019). West Nile virus associated with equid encephalitis in Brazil, 2018. Transbound Emerg Dis.

[10] MS - Ministério da Saúde (2014). Guia de vigilância de epizootias em primatas não humanos e entomologia aplicada à vigilância da febre amarela.

[11] Tolsá MJ, García-Peña GE, Rico-Chávez O, Roche B, Suzán G (2018). Macroecology of birds potentially susceptible to West Nile virus. Proc Biol Sci.

[12] Ross AL, von Matter S, Straube F, Accordi I, Cândido JF (2010). Ornitologia e conservação: ciência aplicada, técnicas de pesquisa e levantamento.

[13] Silva M, Auricchio P (2019). Aves de Teresina.

[14] Sigrist T (2009). Guia de campo Avis Brasilis. Avis Brasilis.

[15] Nicola PA, Kaminski N, La Torre GMD, Barcik JJ, Santos EKMR (2012). Guia de aves do Campus de Ciências Agrárias da UNIVASF.

[16] Sudia W, Chamberlain R (1962). Battry-operated light trap, an improved model. Mosq News.

[17] Consoli RAGB, Lourenço-de-Oliveira R (1994). Principais mosquitos de importância sanitária no Brasil.

[18] Forattini OP (2002). Culicidologia médica.

[19] Kuno G (1998). Universal diagnostic RT-PCR protocol for arboviruses. J Virol Methods.

[20] Catenacci LS, Ferreira M, Martins LC, De Vleeschouwer KM, Cassano CR, Oliveira LC (2018). Surveillance of arboviruses in primates and sloths in the Atlantic Forest, Bahia, Brazil. Ecohealth.

[21] Lanciotti RS, Kerst AJ, Nasci RS, Godsey MS, Mitchell CJ, Savage HM (2000). Rapid detection of West Nile virus from human clinical specimens, field-collected mosquitoes, and avian samples by a TaqMan reverse transcriptase-PCR assay. J Clin Microbiol.

[22] MS - Ministério da Saúde (2022). Guia de vigilância em saúde.

[23] Penazziová K, Korytár L, Pastorek P, Pistl J, Rusnáková D, Szemes T (2021). Genetic characterization of a neurovirulent West Nile virus variant associated with a fatal great grey owl infection. Viruses.

[24] Pérez-Ramírez E, Llorente F, Jiménez-Clavero MA (2014). Experimental infections of wild birds with West Nile virus. Viruses.

[25] CDC (2025). West Nile and dead birds. Centers for Disease Control and Prevention.

[26] Chalhoub FLL, de EM, Duarte BH, de Sá MEP, Lima PC, de Oliveira AC (2021). West Nile virus in the State of Ceará, northeast Brazil. Microorganisms.

[27] Melandri V, Guimarães AE, Komar N, Nogueira ML, Mondini A, Fernandez-Sesma A (2012). Serological detection of West Nile virus in horses and chicken from Pantanal, Brazil. Mem Inst Oswaldo Cruz.

[28] Morel AP, Webster A, Zitelli LC, Umeno K, Souza UA, Prusch F (2021). Serosurvey of West Nile virus (WNV) in free-ranging raptors from Brazil. Braz J Microbiol.

[29] Ometto T, Durigon EL, de Araujo J, Aprelon R, de Aguiar DM, Cavalcante GT (2013). West Nile virus surveillance, Brazil, 2008-2010. Trans R Soc Trop Med Hyg.

[30] Vicente MO, França FGR, Araujo HF (2024). Abundance, temporal variation, and microhabitat use of the house sparrow, Passer domesticus (Passeriformes Passeridae), in urban and anthropogenic environments in northeastern Brazil. Zoologia.

[31] Ferreira MC (2017). Geographic distribution in Brazil and reproductive parameters of native and introduced sparrows (Passer domesticus).

[32] Santos MP, Santos MD (2004). Bird communities in two vegetation physiognomies of the Caatinga in the State of Piauí, Brazil. Ararajuba.

[33] Caten HT, de Oliveira JP, Pascotto MC (2007). Anais do VIII Congresso Brasileiro de Ecologia.

[34] Hufnagel L, dos Santos NB, Regolin AL (2016). Curso de Campo da UFMG; 2015; Belo Horizonte, Brasil.

[35] da Cunha FCR, de Vasconcelos MF (2009). Birds attracted by the vocalization of the Ferruginous Pygmy-owl Glaucidium brasilianum (Aves Strigidae). Rev Bras Ornitol.

[36] de Barros FM (2011). Área de vida, uso e seleção de habitat pela corujinha-do-mato Megascops choliba (Strigiformes: Strigidae) em uma área de cerrado na região central do Estado de São Paulo.

[37] Roda SA, Pereira GA (2006). Distribuição recente e conservação das aves de rapina florestais do Centro Pernambuco. Rev Bras Ornitol.

[38] Santos WM, Rosado FR (2009). Dados preliminares da biologia do gavião-carijó (Rupornis magnirostris, Gmelin, 1788) na região noroeste do Paraná. Rev Agronegócio Meio Amb.

[39] Lignon JS, de Souza Júnior P.Souza EC.Monteiro SG.Pinto DM (2021). Achados parasitológicos em gavião-carijó (Rupornis magnirostris) (Accipitriformes Accipitridae) no Pampa Gaúcho-Uruguaiana, RS, Brasil. Sci Anim Health.

[40] Valim MP, Serra-Freire RT, Fonseca MA, Serra-Freire NM (2004). Níveis de enzootia por ectoparasitos em amostras de rolinha [Columbina talpacoti (Temminck, 1810)] no Rio de Janeiro, Brasil. Entomol Vect.

[41] De Souza VB, Amâncio S, Melo C (2007). Anais do VIII Congresso de Ecologia do Brasil.

[42] Azevedo RA (2024). A avifauna da lagoa do horto florestal, região Amazônica, Zé Doca, Maranhão, Brasil.

[43] Cestari C, Pacheco JF (2010). Aves Emberizidae Coryphospingus pileatus (Wied 1821) a new gathered bird species to São Paulo State and evidences of southern geographic expansion in Brazil. Check List.

[44] ICMBio (2025). Arenaria interpres. SALVE.

[45] Petry R, Peter ÂS, Guadagnin DL (2006). Avifauna do Rio Grande do Sul e doenças emergentes conhecimento atual e recomendações para a vigilância ornitológica da Influenza Aviária e da Febre do Nilo Ocidental. Rev Bras Ornitol.

[46] Catenacci LS, Silva MC, Tajra FS, Barbosa MEC, Nogueira TS, Lima TET (2022). Bases conceituais históricas e registro epidemiológico da Febre do Nilo Ocidental no Piauí. Bol Observ Epidemiol.

[47] Angenvoort J, Brault AC, Bowen RA, Groschup MH (2013). West Nile viral infection of equids. Vet Microbiol.

[48] Paré J, Moore A (2018). West Nile virus in horses - what do you need to know to diagnose the disease. Can Vet J.

[49] Pauvolid-Corrêa A, Morales MA, Levis S, Figueiredo LTM, Couto-Lima D, Campos Z (2011). Neutralising antibodies for West Nile virus in horses from Brazilian Pantanal. Mem Inst Oswaldo Cruz.

[50] Pauvolid-Corrêa A, Campos Z, Juliano R, Velez J, Nogueira RMR, Komar N (2014). Serological evidence of widespread circulation of West Nile virus and other flaviviruses in equines of the Pantanal Brazil. PLoS Negl Trop Dis.

[51] Silva JR, de Medeiros LC, dos Reis VP, Chávez JH, Munhoz TD, Borges GP (2013). Serologic survey of West Nile virus in horses from Central-West, Northeast and Southeast Brazil. Mem Inst Oswaldo Cruz.

[52] Weber MN, Mosena ACS, Baumbach LF, da Silva MS, Canova R, dos Santos DRL (2021). Serologic evidence of West Nile virus and Saint Louis encephalitis virus in horses from Southern Brazil. Braz J Microbiol.

[53] Nunes JPN, Reis LAM, Freitas MNO, do Nascimento BLS, das Chagas LL, da Costa HHM (2023). First isolation and genome sequence analysis of West Nile virus in mosquitoes in Brazil. Trop Med Infect Dis.

[54] Moura S, Pessoa F, Oliveira F, Lustosa AH, Soares C (2012). Animais silvestres recebidos pelo Centro de Triagem do IBAMA no Piauí no ano de 2011. Enciclopédia Biosfera.

[55] Vidaña B, Busquets N, Napp S, Pérez-Ramírez E, Jiménez-Clavero MÁ, Johnson N (2020). The role of birds of prey in West Nile virus epidemiology. Vaccines.

[56] Bayeux JJM, Silva ASG, de Queiroz GA, Santos BSAS, Rocha MN, Rehfeld IS (2019). Epidemiological surveillance of West Nile virus in the world and Brazil. Braz J Vet Res Anim Sci.

